# Chinese and Indian higher education students go abroad: listening to them to determine what their needs are

**DOI:** 10.1007/s11233-021-09078-0

**Published:** 2021-10-20

**Authors:** Adriana Perez-Encinas, Jesus Rodriguez-Pomeda

**Affiliations:** 1 Department of Business Organization, Universidad Autónoma de Madrid, Madrid, Spain; 2 Research Institute on Higher Education and Science (INAECU), Madrid, Spain

**Keywords:** International students, Needs, China, India, University services

## Abstract

This paper voices the opinions of international students’ from China and India, and highlights the intentional process of integrating their perceptions of internationalization into a strategic service delivery plan. Data on those perceptions were analysed using a probabilistic model. We clustered 766 international students’ opinions into categories that enabled us to determine the main ideas that constituted their perceptions. The findings enabled us to draw comparisons between two major sending countries and to formulate a series of recommendations for stakeholders in higher education institutions that receive Chinese and Indian students, as well as for policymakers. Primary differences relate to factors such as learning and internship opportunities for Chinese students and service provision for Indian ones. In conclusion, this study offers the next step in the analysis of Chinese and Indian international students’ needs providing with an innovative way of determining students concerns with a view to empowering them within the internationalization process of higher education institutions.

## Introduction

The growing movement of international students all over the world affects the environment of higher education institutions (Altbach, Reisberg & Rumbley 2009). This paper analyses Chinese and Indian (hereafter C&I) students’ perceptions of their needs when going abroad. China and India are two major sending countries of international students (OECD, 2018). Although these countries have growing numbers of international students going abroad, there is a lack of needs analyses of these higher education students’ perceptions of internationalization in the literature (Altbach and Bassett, 2014; De Wit, Hunter, Howard & Egron-Polak, 2015). The purpose of this paper is therefore to reflect on the needs of C&I students when they go abroad and the services they demand. The reason for this analysis is that internationalization is a process that should encompass students’ voices and international strategies within institutions to keep them active to survive in a changing environment (Kelo et al., 2010; Authors, 2018a). Therefore, in some cases, there is a need to challenge current beliefs and practices in higher education institutions in order to effectively serve and empower international students, especially when they have to deal with extreme cultural differences between their country of origin and the host country.

Data on C&I higher education students’ perceptions were analysed using a probabilistic model that is being increasingly utilised to analyse big datasets (Blei, 2012). It is called a *topic modeling algorithm*, which allows for the elaboration of *probabilistic topic models* in order to discover the main themes latent within collections of words (Brett, 2012). The results, which comprised 766 international students’ opinions – from China and India – were clustered into categories that made it possible to determine the main ideas that constitute students’ perceptions.

The resulting findings enabled us to determine the perceptions of C&I higher education students about what they expect to find in the host country, as well as to draw comparisons between two major sending countries (China and India). An improved understandings of C&I students’ needs and perceptions of their stay abroad can enhance the internationalization process of universities, and change the way of thinking and acting in higher education institutions, taking into account the huge numbers of C&I students going abroad. On this basis, we suggest a set of recommendations and actions for officials in charge of the internationalization of universities.

## Factors affecting the mobility of Chinese and Indian students

There are many types of mobile students all over the world, moving from one country to another. *Credit mobility* is predominant on the European continent, although it is not the largest group of mobile students in the world. On other continents, such as Asia and America, *degree-mobility* students predominate (De Wit, 2008; De Wit et al., 2013). In this paper, we focus on degree-mobility students, specifically in two major sending countries (China and India) from which international student flows are dominated by those seeking full degrees (UNESCO 2009). Both countries are considered to have become influential, not only in the global economy but also in the supply of globally-mobile students (Choudaha, 2011; Sun & Guo, 2020). In fact, according to Varghese (2020), the number of Indian students abroad has increased consistently, 5.2 times from 66.7 thousand in 2000 to 305 thousand in 2017, representing an average annual growth rate of 9.4%. With this data, India represents the second largest student-sending country after China.

In relation to factors affecting the mobility of students, we find literature based on push and pull factors that has been developed by several authors along the years. Back in 1993, Cummings compiled a list of push with the following push factors: human resource capacity, financial capacity, trade dependence, facilitating institutions, domestic scarcity of science and technology offerings, dependence on world economy, information scarcity, linguistic isolation, political uncertainty and cultural disposition. The pull factors were: level of assistance received in the host country, imports and exports, immigration, system compatibility, scale and ability to pay. A number of scholars sought to problematize the great dynamism of students’ motivations to carry out their studies abroad (Rivza & Teichler, 2007), as De Wit (2008) illustrates. He identified and condensed the main factors on the basis of earlier studies in a framework of five main categories: educational, political, social, cultural and economic, in order to identify several push and pull factors for outward mobility.

The growing literature on higher education internationalization and globalization (Lee & Stensaker, 2021; Seeber et al., 2016; van der Wende & Zhu, 2016), considers that the increasing global economic integration is the main cause for student mobility.

According to Sun and Guo (2020) the economic globalization has led to the increasing demands for internationalized talents in the labor markets, even when the students’ results after their international mobility are disparate (Costello, 2015; Davis & Knight, 2021; Mikuláš & Jitkab, 2019).

In this line, the ‘push-pull’ model tested in the study by Mazzarol and Soutar (2002) indicates that economic and social forces within the home country serve to ‘push’ students abroad, but the decision to select a host country depends on a variety of ‘pull’ factors. According to Lee and Tan (1984), perceptions of the quality of higher education system offered in the home country, the relative wealth of the home country and the GNP growth rate in the home country also have an impact on the choice of country in which to study abroad. Furthermore, studies from the 1970s also suggest that the student flow is dependent on the wealth of the home country and the degree of development (McMahon, 1992). Mazzarol et al. (1996) later identified six factors that influenced the students’ selection of a country in which to study abroad. These are: (1) the knowledge and awareness of the host country in the students’ home country; (2) referrals and personal recommendations; (3) the cost of living, studies, travel and social events; (4) the environment or climate of the host country; (5) the geographical proximity; and, finally, (6) the social links.

The literature on students’ motivations gathers also empirical studies on multidimensional scales (Aresi et al., 2018). Some scales are specific to Chinese students, as Chirkov et al. (2007) show. These researchers provide convincing evidence on the reasons that Chinese students have for their international experience, showing that the improvement of their future professional opportunities abroad (after completing high quality educational programs) is the key for the mobility decision. Other lines of research on student’s experiences and students’ problems while studying abroad have shed light on issues related to their motivation to enrol in foreign programs. There are studies on social, cultural and linguistic capital gained through international experience (Bótas & Huisman, 2013); on the expectations, experiences and results obtained by Chinese students (Comerio & Walker, 2021; Hu et al., 2021; Sablina, Soong & Pechurina, 2018); on student mobility as an engine of higher education internationalization (Fumasoli, 2021); on the relationship between universities’ rankings and students choice (Hazelkorn, 2015); on international students’ satisfaction after their educational and non-educational (culture, transportation, accommodation, administrative issues) experience (Nada and Araújo, 2018; Huisman et al., 2020); on the reasons why some students go abroad when other ones stay at home (Matthies & Karhunen, 2021); on students’ opinions on their emotions at the host university (Bañegil-Palacios & Sánchez-Hernández, 2018) and, in a wide perspective, on the value of students’ feedback on their educational experience (Williams, 2014).

In relation to authors like Mazzarol and Soutar, there are a series of factors influencing students’ decision to study overseas based on students from Taiwan, India, China and Indonesia. Five factors were identified, related to (1) whether the overseas course was better than the offer in the home country; (2) the difficulty gaining entry in the home country; (3) if the course was not available at home country; (4) a good understanding of the West; (5) and whether the intention to migrate was also a factor.

In the case of Chinese students, the two predominant factors in Mazzarol and Soutar’s (2002) research were: the overseas courses were better than the local ones and the intention to migrate. For Indian students, the main factors were a good understanding of the West and the overseas courses were better than local ones. These findings highlight the importance of understanding international students’ needs by listening to them and offering them a quality experience abroad by providing adequate support services for their specific needs. While in the case of India, students studying abroad seek options that cost less, and enhance career opportunities and returns on investment. They opt for English-speaking countries which offer good international education and job opportunities after studies (Varghese, 2020).

It will be important to identity the factors affecting the mobility of this two big groups as neither all students have same needs (Authors, 2018; Archer et al., 2010) nor all students are the same (Choudaha, Orosz & Chang, 2012).

## Do we listen to them? Empowering international students according to their needs

Empowerment is an intentional, ongoing process centred in the local community, involving mutual respect, critical reflection, caring, and group participation, through which people lacking an equal share of valued resources gain greater access to and control over those resources (Cornell Empowerment Group, 1989). The starting point of any proposal aimed at the empowerment of international students is knowledge of their needs. However, little has been written on students’ needs when they go abroad (Authors, 2017). It is also clear that international students might not always be aware of these needs and thus not adequately prepare for their experience abroad. It is therefore important to investigate their opinions on several aspects and offer the appropriate support services to empower them during their mobility period. According to Zimmerman (2000), empowerment is a psychological process in which individuals think positively about their ability to make changes and gain mastery over issues at the individual and social levels.

In fact, by recognizing and identifying their needs, we can provide them with the appropriate channels that give them the ability to make change, and to look for a better fit between their needs and the services and support offered by host universities. This support can apply to a wide range of activities and services offered to students, such as providing information, arranging accommodation, providing access to dining facilities, offering welcoming activities, or offering academic or language support (Kelo et al., 2010), and also to discover what international students think and say about support services and their needs. It is not easy to determine the best way to meet the needs of international students (American Council of Education, 2016), because international students face differing issues at differing institutions, so they might need a diverse set of support services (Authors, 2016).

University students (in this case, C&I) who have a study experience abroad form an opinion about it based on their perceptions of a multitude of academic, cultural and lifestyle aspects in the host country. Therefore, the perceptions lead to a judgment of value or opinion regarding the greater or lesser coverage of their needs of different types in that country and in a specific university. When in their reports they emphasize the presence or absence of a certain aspect, they are recording how the country and the university meet the needs that students have had during their stay abroad. Sometimes they present their suggestions as “wishes” so that the host country and university better meet the needs of the foreign student. It should be borne in mind that, given the relative cultural homogeneity of Chinese students -on one side- and Indians -of another- when facing an international experience in a country culturally far from them, these wishes represent a useful recommendation for countries and host universities offer a better range of services to future C&I students.

Thus, sourcing international student’s opinions is more important than ever, because these students are a key component of the internationalization process of higher education institutions. It seems reasonable that once we have gained knowledge of the needs of the students from China and India (countries with huge numbers of outgoing students (UNESCO, 2009)), we should be able to contribute useful ideas on their empowerment related to the provision of services by host universities. This kind of development could be integrated into universities’ capital intellectual schemes (Machado & Rodriguez-Pomeda 2008).

## Methodology

The method used in this study is widely used to analyse big datasets. It gathers quantitative as well as qualitative information, and avoids researchers’ biases through unsupervised analysis based on Bayesian probability. This method is called a topic modeling algorithm. It allows for the elaboration of probabilistic topic models employed to discover the main themes latent within collections of words (Blei, 2012; Brett 2012). Recent developments have emphasized the potential and appropriateness of this family of models to study higher education issues involving huge amounts of data (Daenekindt & Huisman 2020; Authors, 2018b; Rodriguez-Pomeda & Casani 2016). Probabilistic topic modeling implies the search for a concealed structure emerging from the topics found within the considered sets of data (words, in our case). A topic is a pattern of words that demonstrate a persistent trend appearing together (or closely) in a corpus of texts (Brett, 2012). One of the basic probabilistic topic models is the LDA (Latent Dirichlet Allocation) model (Blei, 2012).

The data came from the large StudyPortals database and their online platform STeXX (www.stexx.eu; see also www.studyportals.com). On STeXX, students are enabled to write about and share their study abroad experiences by reviewing their host universities. This initiative of StudyPortals is supported by a group of well-known international student associations such as AEGEE (Association des Etats Généraux des Etudiants de l’Europe), ESN (Erasmus Student Network), ESTIEM (European Students of Industrial Engineering and Management), and SIU (The Norwegian Centre for International Cooperation in Higher Education). STeXX has the support of the European Commission and the authors have signed an agreement with StudyPortals B.V.to use STeXX data for research purposes only, with a number of restrictions. The initial collection of the main dataset of students’ perceptions on support services in higher education institutions abroad for this research started in 2011 and ended in 2014. Nevertheless, the authors followed up with several data additions in 2018 and 2021 in a continuous contact with StudyPortals B.V. to update the information about the new features of the platform and provide with the most accurate data. In fact, StudyPortals kindly sent us the latest reports for the period January 2014 to July 2021 prepared by C&I students abroad, which are presented additionally to this paper and gather not only a more updated information from C&I students but also reflect the current situation lived by international students abroad due to COVID-19.

### Sample

The StudyPortals database initially used in this article included 73,715 reports written by students from 167 countries. Therefore, to achieve the research objectives, the 766 reports corresponding to C&I students who completed a period of study abroad were selected. Additionally, the reports of students who made their mobility to their own countries were eliminated (in the case of China, including mobilities from/to Macau and Hong Kong, SAR to/from mainland China) and all reviews were checked and processed to exclude internal mobility (within the same country), as well as datasets in languages other than English, and stopwords (common words such as conjunctions or articles that do not add relevant information to the analysis (DiMaggio, Nag & Blei, 2013)). The period covered corresponds to the years 2011 to 2014, with subsequent additions provided by StudyPortals corresponding to 2018. In the final phase of preparing this article, the authors accessed a new database consisting of 20,731 reports corresponding to the 2014–2021 period. After cleaning this database with the same criteria indicated above, a reports’ sample dated from January 2020 to July 2021 was selected to initially analyze the impact that the COVID-19 pandemic has had on the international experiences of these students. Since, unfortunately, the COVID-19 pandemic continues its course, the information collected is preliminary and is being updated as new reports are received from students that allude to it. The effects that such a devastating health crisis will ultimately have on the needs of C&I students abroad can only be determined when the health situation is normalized.

We have developed the topic model for two main ones (one for each country). Like the topics made up of the words collected in the previous models (Tables 1 and 2), the new topics reflect the opinions of Chinese and Indian students (Tables 4 and 5) after their study abroad experience. In other words, it is not about expectations, but about opinions after the international stay.

**Table 1:** The LDA model with 10 topics with data from 2011 to 2014 main defining words (China analysis)

Topic	Topic label	Words within the topic
19	Academic & social environment	good city people university nice study beautiful great environment place experience education life friendly studying live facilities teachers
14	Social & cultural life	learn study life culture friends experience make time enjoy country local learning lot abroad find language social important home
8	Solid teaching	students university courses teaching professors international research lot world program famous information lots class make methods top great advanced
7	Living expenses	expensive month euros living cost rent year student fee live euro accommodation cheap expense tuition food money
16	Learning & internship experience	hard work foreign internship close courses large reasonable mandatory academics give months qualified kinds test healthy teacher extremely
9	Learning spaces	English university speak system skill learning view convenient communication problem required nature lesson equipped cosy Arnhem choosing
3	Winter & money	business school winter cold clothes bring management small weather town money understand negotiation global personally extremely skiing warm
15	Social life	strong support choose months group shows seminars bike public fun free give life depending transport Granada Asian
10	Cultural offering	cities France travel Brussels Flensburg Germany traditional Italy small place system transportation integration areas expansive UK range wide Belgium
2	Transportation & Social Life	opportunities price offers time transportation regret public euro room evenings bars hope Germany drinks wasnt fishing generally speed

**Table 2:** The LDA model with 10 topics with data from 2011 to 2014 main defining words (India analysis)

Topic	Topic label	Words within the topic
7	What a good university is	university students good city study student research education international great facilities lot place country recommend campus quality work
0	Social & Cultural life	time life make learn people experience friends language things future studies advice exchange culture career spend social enjoy travel
10	Living expenses	living expensive cost month accommodation food live euros expenses fee fees tuition cheap year euro costs compared
1	Solid teaching	teaching courses good methods students choose academics research practical oriented method professors technical interest provide exposure taught business staffs
4	Service provision	India Germany school accommodation Europe cultural cheaper find heart provided institutions perfect products understand Indian rupees Mumbai comparatively
12	Finance help	institute wide present practical range qualified government college reputed Italy doubts educational subject activities financial simulations road solely ups
13	Accommodation	graduate housing PhD expensive work countries find extremely option regular culture prefer post important subjects hard house apartments
9	Academic life	world-class exposure universities major makes fields cultural alba places members professors visiting terms rooms maximum industry experiences events
18	Academic learning	interested science computer thesis department thing security join dream back started explain areas involved ended slides care
15	City offerings	city people Athens cities French mind welcoming ideas love open days warm week lovely inter Delhi strongly information issue

### Analysis

With all the reviews, we elaborated a probabilistic topic model. On this model, we propose a set of labels that – in our view – represented the raw information contained in the C&I students’ reviews. In the tables below, we show our proposed label codes indicated by the resulting groups of words from each topic. The resulting probabilistic topic model involves an acceptable interpretation by the authors of the semantic coherence of all the words related to a specific topic (Chang et al., 2009a, b; Authors, 2018b). The code name ‘proposed label’ applies to a dataset of words related to a topic, as can be seen in the findings table.

First, we obtained a probabilistic topic model based on a Latent Dirichlet Allocation (LDA) with 10 topics capable of generating the whole set of student reports with their comments and views on their experience abroad in relation to diverse services. The aim of any topic model is to draw a coherent argument about large series of textual data. Specific algorithms (like LDA) allow the extraction of the main topics underlying a set composed of a huge number of words. LDA is a not-supervised method to obtain those topics, so the researchers do not impose any constraints in the search for topics. Therefore, there are no biases in the topics found. Topics are “bags of words” that describe a feature of the phenomenon investigated. LDA gathers some topics (constituted by words ordered by its frequency within each topic) that must be interpreted by the researchers. The reason is that topics are considered as hidden variables representative of latent dimensions of the dataset. So, topic modeling is an inductive research strategy. Researchers feed the model with the number of topics that fits well with the amount of words employed to run the model. The literature suggests that ten is an appropriate number for our dataset. The following step is to write a descriptive label for each topic. These labels result from researchers’ interpretation taking into account the semantic coherence of the words gathered by the model within a specific topic (Chang et al., 2009a, b). Previous knowledge on the research issue drives the process of topic characterization, naming, and contextualization (Ramage et al. 2009). But this process is not univocal, and, usually, the most frequent words within a topic are employed to describe it (Ramage et al., 2009). The labels were derived by the authors from the results provided by MALLET (a package for statistical natural language processing, classification, clustering, topic modeling, and other machine learning applications to sets of texts ((McCallum, 2002)). The proposed LDA model is presented in Table 1 below. Thus, the labels are based on the authors’ interpretation in light of the relevant literature about international student mobility and the resulting words from the model (Ramage et al., 2009).

The following tables (Tables 1, 2, 4 and 5) show the main words in each of the 10 topics, and the labels proposed for those 10 topics.

## Results

The results are presented in Tables 1 and 2 below. The first table shows the results or topics from the analysis of the Chinese international students’ comments. Those topics are ordered by weight in the composition of the whole set of reviews, and therefore also by importance: The topics clearly state that Chinese students going abroad focus mainly on the academic and social environment. The cultural life is also an important factor to choose a destination abroad but more important is the relation to the academic world and what we called “solid teaching”. The money and living expenses is the fourth concern we have found after running our analysis, followed by the opportunities of learning experiences and internships.

When comparting with Indian students results, we can see in the following table the results from the Indian international students’ comments. From their comments, we deduced that the reputation of a university and how good the university is, as a key issue to select a destination. This concern is followed by the social and cultural life and on third place, the living expenses, how expensive the full cost is going to be. The quality of teaching is not occupying a high position but still is in the top five concerns, followed by an interesting one that Chinese students did not mention in their priorities, like is the service provision in the host institution that relates to next concerns on accommodation issues or academic life.

The results after analysing the Chinese and Indian international students’ comments on the major problem areas concerning host university support services (related to their needs) yielded the following results based on weights and connection strengths (see Table 3).

**Table 3:** Common factors categorized by label to compare Chinese vs. Indian mobility students (data 2011–2014)

Analysis of Chinese students’ reviews	Analysis of Indian students’ reviews
Academic & social environment	What a good university is
Social & Cultural life	Social & Cultural life
Solid teaching	Living expenses
Living expenses	Solid teaching
Learning and internship experience	Service provision

A deeper examination of the results demonstrates the relevance for Chinese students of the academic and social environment. Here the students’ needs axis are the quality of the whole environment (‘city, people, university’), the development of an enjoyable experience abroad (‘life, friendly, live, facilities’), and the distinction of the academic framework (‘good, study, education, teachers’). For the Indian ones – even when there are some similarities with their Chinese peers – a different set of words arise relating to their main concerns about their experience abroad. They underline in their reviews ideas related to a global academic environment (‘research, international, facilities, country, recommend, campus, quality, work’).

‘Social and Cultural life’ is the second relevant point that both C&I students write about in relation to their perceived needs when they study abroad. The topics labelled ‘Living expenses’ and ‘Solid teaching’ occupy similar positions in the ordered relationship of Chinese and Indian students’ needs.

In Table 3, also enables us to identify differences and similarities between the Chinese and Indian students’ concerns when they go abroad. Both groups paid attention to academic issues and social life and culture. However, while the Indian students focused more on the reputation of the host university, for example when choosing institutions in English-speaking countries as they are considered to be good institutions (Varghese, 2020), the Chinese students combined the reputation and academic environment with the social offerings. This is in line with the findings from Mazzarol and Soutar (2002) where Chinese students focused on academics, such as the overseas courses that they are different that in their home country. Living expenses were identified by all the groups and, in the case of the Chinese and Indian students, this topic was among the primary common factors. In the fifth position, the Chinese students agreed on aspects related to learning experiences, as well as on internships possibilities and offers. By contrast, the Indian students focused more on service provision during their stay.

An interesting conclusion derives from the magnitude of the topic designated ‘Service provision’ in the analysis of Indian students’ reviews of their experience abroad. Indian students talk about places (‘India, Germany, Europe, Mumbai’), human touch (‘heart, understand’), price-quality relationship (‘cheaper, rupees, comparatively’), as well as some of the main elements required for a good service provision (‘accommodation, cultural, provided, institutions, products’).

Conversely, for Chinese students abroad the last one of the five main concerns about their needs is the topic ‘Learning and internship experience.’ The first words in this topic are ‘hard’ and ‘work’, signalling their attitude towards an abroad experience that can offer them a differential qualification compared with their fellow citizens who remain in China. Another relevant words in the topics are ‘internship’, ‘mandatory’, ‘academics’, and ‘qualified.’ It appears that Chinese students try to obtain the best from their international experience. China’s internal job market dynamics and private schemes to finance the experience abroad should perhaps be related to this conclusion. To summarize, the two groups shared common perspectives and needs. The first shared one was the social and cultural life aspect, together with solid teaching and living expenses. Both groups of international students also commented on academic issues and social environment as primary concerns. The main difference appears in the last factor; the Chinese students commented more on their learning and internship experience, and the Indian students on services provided.

Additionally to the previous data, we have also analysed reviews from 2014 to 2021 with a special focus on the impact of the COVID-19 pandemic from January 2020 to July 2021 on Chinese and Indian students. Results are shown in next Tables (4 and 5).

**Table 4:** The LDA model with 10 topics with data from Jan’20 to Jul’21: main defining words (China analysis)

Topic Topic label	Words within the topic
8 Academic life & living concerns	university school life international degree united resource big coming interdisciplinary restaurants information cutting-edge tons worth understand panic financial helpful
5 Teaching issues	united teachers nice cultural resources love deity score work perspectives interdisciplinary care technology addition trips things
0 Social life	experience good atmosphere short day well-designed professors lovely program children
6 Covid academic experience	students activities covid love suggestions problems solve excellent teaching include living support cultures surrounding fortunately challenges online
4 Social & Cultural life	friendly appreciation hard environmental backgrounds working integrate English activities opportunities experience companions backers act ocean study
2 Covid learning experience	school center satisfying give teaching clinic field understand ideas students speak covid organized academic peak atmosphere people intellectual social
3 Covid & facilities	student infrastructure solar program local chinese group materials covid school guides computer states practices exposure granted learning master great
9 Social & cultural life	united environment research fell related enter learn healthy customs local students system science climb bridge university
7 Research issues	teachers experiences participated famous culture academic nourishment studying research interesting labs shared people nice imperial property publishing market international
1 Living concerns	love appreciated facilities course word experience human states highly stressful functioning industries learning progress

**Table 5:** The LDA model with 10 topics with data from Jan’20 to Jul’21: main defining words (India analysis)

Topic Topic label	Words within the topic
1 What a good university is	good faculty study modules techniques data training pursue plan helpful urban libraries setting networked don’t ready lot nest
9 Career prospects	experience career students study partner science working travel love carrier field year area haven’t difficult full business date home
4 Solid teaching	good world learning core covers option perfect programme introductory phd industry management areas superb persistence designed instance global local
0 Covid learning experience	covid start experience visualization data explored daily psychology distance key top staff studying travelling outbreak internship due number current
5 Learning experience	university regret academic machine school datascience poor college rural business strict marking thing cooperative things open situation English easy-going
2 Academic life	content university studies rewarding programme tourism background rural reached started thesis complete unable learn unrest covid make
3 Academic teaching life	taught time programs books curriculum coming lot affected mindful effective virus health public quick webinars give good enthusiastic
8 Social & Cultural life	great time honestly idea sem travel bachelors named traveled choice requirements home made exposure birds teaching internship practice hands
7 Covid academic experience	online year college shine train spend staff situation leave based teaching ongoing covid due hey parts learner enjoyed
6 Academic curriculum	feel knowledge master research international excellent radiologist programme aspirants sort education friendly semester gain helped opportunity great study

For Chinese students is worth noticing that topics related to academic issues and COVID learning experience are key during this period. This data are related to the previous set of reviews, where also Chinese students expressed their concerns on academic life. Nevertheless, in this last wave of data from January 2020 to July 2021, the amount of reviews and comments on academic life increased significantly and the COVID-19 scenario has been added to the picture. Less attention has been paying to social and cultural life and finances or living expenses were not even mentioned in this set.

Next table reflects the main defining words for Indian students from January 2020 to July 2021. Main findings for this group and data set agree with the previous data on Indian students, being the most comments and defining words those related to the label “what a good university is”. Social and cultural life are not so significant in the analysis and living expenses or finances are not mentioned as it happened with the case of Chinese students. On the other hand, academic life plays a more important role and more concerns on COVID learning experiences arose.

All in all and in relation to the last wave of analysed data, we see a trend for both groups of more focused on academic life and on the learning experience (like for example teaching issues or curriculum). These new labels arose with the data since the COVID-19 pandemic. We also notice less emphasis in social and cultural aspects and even less in financial topics.

### Recommendations

The results presented in the previous section indicate key factors about international C&I student needs and experiences abroad. Now we deal with some suggestions based on them with a view to attaining an enhanced management of international student flows arising from China and India, and also for the empowerment of C&I students abroad. The results obtained from the topic models show, in order of importance (given by the relative probabilities of the presence of each topic in the set of reports analyzed), what are the opinions of C&I students with respect to the higher or lower meeting your needs in host countries and universities. This method of directly collecting opinions from Chinese and Indian students about their needs abroad is - to our knowledge - a novelty in the literature that provides a deep capture of highly relevant information so that host countries and universities can offer a better service to students who, coming from these countries, will welcome in the future. Therefore, the results achieved allow the elaboration of well-founded recommendations that may or may not materialize (according to the actions of the host universities) in a specific list of actions aimed at increasing the satisfaction of students from C&I. It should not be forgotten that, with data prior to the COVID-19 pandemic (post-pandemic data is still uncertain and incomplete), such countries had an extremely prominent presence in the global pool of university student mobilities.

Taking into account the breadth of the expressed needs (and their connections with several university management areas, such as teaching services, curricula updating, partnerships with universities’ external stakeholders, and so on) the very first recommendation for the host universities is to develop an integrated vision of the provision of services for incoming C&I students. A strong international host university orientation is also needed to establish the right set of relationships with these students. Thus, the universities should combine specific units and people to offer the complex services demanded by C&I students. The reason is that these students have specific mindsets related to their respective cultures of origin, which are completely different from those maintained in Western universities, which appear as the main hosts in the STeXX database. Results from both groups indicate that in a “normal scenario” without a pandemic situation the social environment and the social and cultural life are important when choosing a destination country to study. In this respect, one recommendation for HEIs in respect to the social and cultural offerings could be integrated in the service provision that the HEIs offered as one more service, so the attraction of these groups of students can be higher.

On a different note, Chinese students gave a higher priority to the courses and teaching in the host institution. To this respect, it will be recommended that once that integral international view is established within the host universities, it is advised that the universities should enact efficient incentive schemes for their faculties in order to strengthen their attitudes to teaching innovation.

Another suggestion is to strengthen their partnerships with firms and other organizations that are willing to accept international students for their internships. C&I host countries’ governments could facilitate agreements with local suppliers aimed to offer C&I students better returns. It could be interesting that local governments in the C&I students’ host cities develop specific cultural and social programs for those students; so, students should enjoy enhanced experiences in the host cities. As clearly seen in the results, learning practices and experiences are a key decision factor for Chinese students.

Host universities should also buttress the administrative services that they provide to C&I students to avoid misunderstandings and errors, because those problems harm the students’ perceptions of the quality of the universities and could also have harmful academic consequences for the students (such as delays in finalising their programs).

Exceptionally, other variants should take into account when defining the needs of international students and should take into account each context. As the current COVID-19 pandemic is affecting higher education institutions and mobilities, deeper analyses should be carry out to understand the needs of each of students in every context. According to the results of the last year analysed for C&I, we have seen that a higher percentage of comments from those students centred on academic issues and less on social and cultural aspects, none the financial aspects has been commented.

The first results collected within the pandemic (from January 2020 to July 2021) indicate that “academic life and living concerns”, “teaching issues”, “social life”, “Covid learning experience”, and “social and cultural life” are the five main topics in the Chinese students’ reports. For the Indian ones, “what a good university is”, “career prospects”, “solid teaching”, “Covid learning experience”, and “learning experience” are the main topics. The general picture, which emerges from the analysis, is that more changes appear within the Chinese students’ opinions. Academic, living concerns and teaching issues are the themes that gain relevance. However, some of the words that define the corresponding topics appeared also in the issues obtained with the 2011–2014 sample. A brand new topic (within Indian as well as Chinese students’ reports) is “Covid academic experience” (that captures the specificities of the pandemic period). Given the pandemic shock, our findings should not be over-interpreted. It is clear that our findings on the January 2020 to July 2021 period are based on a scarce number of reports. Therefore, more data are needed to confirm the prevalence of the observed changes in the topics.

Next table collects information of suggested actions or recommendations for this specific population groups, based on the previous results on C&I students going abroad. In our view, following these suggestions would assist in enhancing the empowerment of higher education C&I students when they go abroad (Table 6).

**Table 6:** Suggested actions for universities

Label	Suggested actions
What a good university is	To improve teaching techniques; reduce students/professor ratio; to enhance the university experience; to offer comprehensive information; to offer internationalization at home
Living expenses	To build facilities and alliances to offer a good value-for-money relationship
Solid teaching	To design and develop updated and internationalized curricula; to encourage good teaching techniques; to apply better student evaluation schemes
Expensive country	To implement deals with public agents to smooth students costs, considering that the host country benefits from mobility students’ expenses
City offerings	To develop integrated networks for the full enjoyment of all the city aspects (cultural, social)
Learning & internship experience	To partner with companies to offer internships abroad during and after the studies of international students. To enhance the learning experience by including academic staff in the internationalization process of universities.
Service provision	To provide support services that cover international students’ needs. To periodically assess trends on support services and market demands. To allocate resources to fulfil the expectations of international students.

## Conclusions

This study offers the next step in the analysis of C&I international students’ needs. It also provides an innovative way of determining students concerns with a view to empowering them within the internationalization process of higher education institutions. The method clarifies the gaps identified by the students after their international experience. The topics arising from the whole set of students’ reports are composed of words that explain the keys of C&I students’ international feel. It is important to fulfil the expectations of international students with the services offered in order to increase their satisfaction with their stay abroad (Authors, 2017). Five major areas of interest were identified among C&I students. The common ones were related mainly to academic life and university reputation, and social and cultural life. The primary differences were related to factors such as learning and internship opportunities for Chinese students and service provision for Indian ones. Nevertheless, when we analysed data from the last year 2020 to 2021, both groups of students have similar concerns mostly related to the academic issues. Data from January 2020 to July 2021 suggest that teaching issues has gained momentum within the reports analysed from Chinese students, and that career prospects deserve more relevance considering the reports analysed from Indian students. We find that issues related to the Covid academic experience are relevant for Indian as well as for Chinese students.

Our findings point to the main concerns that delimitate the perceptions of C&I higher education students of the academic services and social life found in the host universities. It is important for host universities to deliver high quality academic services to C&I students, as these services boost students’ perception of universities as world-class ones.

A careful consideration of our study’s results could inform a set of extended recommendations aimed at grounding efficient actions boosted by policymakers and universities’ managers responsible for empowering C&I international students and to start changing the way of thinking and acting in higher education institutions. Consequently, the student experience abroad of C&I students could attain higher satisfaction levels generating a virtuous circle with the attraction of higher number of C&I students in the following periods.

## Limitations and further research

This study revealed how the results changed due to the COVID-19 pandemic. In this sense, it will be advisable to analyse data post-pandemic to see how it has been affected to the in the global pool of university student mobilities and compare it with other countries.

Another interesting study could be an analysis of the reasons for the differences found between Chinese and Indian students in their evaluation of their respective needs. For instance, for Indian students ‘Service provision’ is a relevant concern. Perhaps the longer historical flows of Indian students to foreign countries (compared to their Chinese peers) should explain this result. Thus, a broader research will be needed to draw broader suggestions and recommendations to these population groups. This promising research avenue could be analysed with the new family of methods developed under the computational social sciences umbrella. Within them, probabilistic topic models constitute a strong tool to deal with the huge amounts of data generated by the large international student flows, and are increasingly used in research within the higher education field.

## Data Availability

Data subject to third party restrictions: The data that support the findings of this study are available from StudyPortals, B.V. (https://studyportals.com). Restrictions apply to the availability of these data, which were used under licence for this study. The data used in this study were available to the authors with the permission of StudyPortals, B.V.
